# Digital Medical Cannabis as Market Differentiator: Second-Generation Artificial Intelligence Systems to Improve Response

**DOI:** 10.3389/fmed.2021.788777

**Published:** 2022-01-24

**Authors:** Yaron Ilan

**Affiliations:** ^1^Faculty of Medicine, Hebrew University, Jerusalem, Israel; ^2^Department of Medicine, Hadassah Medical Center, Jerusalem, Israel

**Keywords:** artificial intelligence, cannabis, digital health, artificial intelligence (AI), delta9-tetrahydrocannabinol (THC), cannabidiol (CBD)

## Abstract

Legalized use of cannabis products and the rising interest in their therapeutic benefits have opened up new opportunities for therapy and marketing. However, the marked variability in formulations, administration modes, therapeutic regimens, and inter- and intra-subject responses make the standardization of medical cannabis-based regimens difficult. Legalization has made the cannabis market highly competitive and lowered the revenue margins. This study reviews some of the challenges in medical cannabis use and difficulties in standardizing its therapeutic regimens that hinder maximizing its beneficial effects. The development of tolerance toward cannabis and low adherence to chronic administration further impair its long-term beneficial effects. Digital medical cannabis is a cannabis product controlled by a second-generation artificial intelligence (AI) system that improves patient responses by increasing adherence and dealing with tolerance. Second-generation AI systems focus on a single patient's outcome and deal with the inter- and intra-subject variability in responses. The use of digital medical cannabis is expected to improve product standardization, maximize therapeutic benefits, reduce health care costs, and increase the revenue of companies. Digital medical cannabis offers several market differentiators for cannabis companies. This study presents a model for promoting the use of digital medical cannabis and presents its advantages for patients, clinicians, health care authorities, insurance companies, and cannabis manufacturers. Ongoing trials and real-world data on the use of these systems further support the use of digital medical cannabis for improved global health.

## Introduction

Cannabis is the most commonly used illicit drug worldwide. The active constituents of the product were described several decades ago (1). The changing legal landscape and rising interest in its potential therapeutic utilities have opened new opportunities for therapy (2). Multiple cannabis products are increasingly used in countries that have legalized their use. The large variety of formulations, and different administration modes, therapeutic regimens, and inter- and intra-subject responses have made it difficult to standardize the therapy and maximize the therapeutic potential of these products. In addition, cannabis companies face increased competition and lower revenue margins, further impacting development in the field.

This study reviews some of the challenges in developing and marketing cannabis products. The potential of second-generation artificial intelligence (AI) systems for maximizing the personalized benefits of these therapies is described. Furthermore, the paper presents the development of digital medical cannabis as a market differentiator that improves patient responses and increases the drug's market share.

## Challenges in Medical Cannabis Use: Difficulties in Therapy Standardization Hinder Maximizing Its Beneficial Effects

The increase in strength of THC-plants composition is related to legalization, globalization, pharmaceutical-related factors (3, 4). The increased use of medical cannabis for multiple diseases and legalization of its use in many countries have led to the increase in variety of products available. Multiple cannabis plants and strains and different growth modes, extracts, final formulations, and delivery methods are being promoted. In addition, distinct batch-to-batch variability further complicates the standardization of products (5–9).

The legalized cannabis market is dominated by high delta9-tetrahydrocannabinol (THC) cannabis flower and shows growing expenditures on extracts. Higher-strength THC has become increasingly available after the legalization of cannabis. As for cannabis flowers, both THC and non-psychotomimetic cannabidiol (CBD) are associated with higher per-gram prices. Traditional cannabis flowers account for most of the spending (66%), with increased market share of extracts for inhalation, representing 21% of sales (10). A recent study of Washington state's legalized cannabis market showed considerable diversity in terms of product and pricing. While edibles accounted for a modest share of consumer spending, extracts for inhalation comprised a larger and heterogeneous market segment (11). Concentrate users choose higher-strength cannabis and reveal higher cannabis use disorder symptoms. Frequent concentrate use has higher risks than the use of flower forms (12).

A study comparing two strains of cannabis, one with 8% THC and 16% CBD (THC+CBD), and the other with 17% THC (CBD) concentration but no CBD (THC), showed that the THC+CBD strain is associated with lower cannabis craving, subjective intoxication, and circulating cytokine levels. The data suggested that patients may self-titrate their cannabis use based on cannabinoid concentration (13).

Cannabis is a highly personalized medicine, which needs to be titrated up. The pharmacokinetics of most cannabis products is not known. The major cannabinoids are substrates for numerous metabolic enzymes, such as cytochrome P450 metabolizing enzymes (6). The PK of oral THC shows marked variability, with differences between formulations, for example, higher variability in baked goods and oil forms (14). The preparation method significantly impacts the final product characteristics (15).

Besides the variability in products and delivery modes, multiple host factors impact the overall response rate. A recent study of 4,000 subjects consuming cannabis at least once in the past year classified the subjects into four groups based on number of use days per year as follows: infrequent users (<11 days), occasional users (11–50 days), regular users (51–250 days), and intensive users (>250 days) (16). Considerable differences could be found in annual cannabis consumption across countries (16) and in inter- and intra-subject variability for the pharmacokinetics of cannabis formulations (17). The host genetic background also affected the product effects (18–20), while fasting affected the pharmacokinetics of the active compounds in cannabis oil extracts (21). Consumer preferences are a major factor for the selection of formulations to be used. Medical cannabis users with chronic pain show distinct differences in cannabinoid use and administration mode preferences. Gender, use intention, and past experience are some of the relevant parameters (22).

Clinicians also fail to adhere to cannabis prescription guidelines, further impacting the difficulty in standardizing therapeutic regimens. In a retrospective case series analysis, physicians did adhere to the cannabis prescription guidelines in pre-existing cannabis prescriptions for over 85% of users who were prescribed cannabis products for pain and posttraumatic stress disorder (23).

These examples indicate the challenges in medical use of these products for both caregivers and patients. Commonly, the right formulation, preferred dose, and delivery mode are identified and appropriate therapeutic regimens are selected on a trial-and-error basis, rather than on validated data. Thus, physicians and patients find it very difficult to maximize the beneficial effects of these products.

## Tolerance Toward Cannabis Impairs the Long-Term Effects of Cannabis Products

Partial and complete loss of response to chronic medication is a common problem with multiple drugs (24) when regular cannabis users develop tolerance to its effect (2). Cannabis tolerance models imply neurobiological or behavioral adaptation following repeated cannabis exposure (25). A review of studies examining the single or repeated cannabis administration effect as a function of previous exposure showed the acute single cannabinoid administration effect less acute in chronic users compared to non-regular users (3). Repeated cannabinoid administration is associated with decreased effectiveness upon repeated exposure, mainly for cognitive functions. Regular exposure is associated with reduced acute intoxicating, psychotomimetic, and cardiac effects, or partial tolerance. Some chronic users showed full tolerance, with complete absence of acute effect (3, 26).

The development of tolerance to around-the-clock oral synthetic THC use was evaluated in daily cannabis smokers over a period of 6 days. The morning subjective intoxication ratings increased from day 1 to day 2, and then declined over days 4 and 6. The morning THC dose increased intoxication ratings on day 2 but showed less effect on days 4 and 6, a pattern consistent with tolerance. Six days of around-the-clock oral THC use produced tolerance to subjective intoxication but not to cardiovascular effects (27).

The mechanisms of this neuroadaptation underlying cannabis tolerance are unclear. The downregulation of CB1 receptors in chronic users is associated with dopaminergic output normalization from the ventral tegmental to mesolimbic circuit area. This is associated with reduced impairment during acute exposure. Neuroadaptations are absent in occasional users, who reveal strong increases in dopamine and glutamate levels in striatum, loss of functional connectivity within the mesolimbic circuit, and neurocognitive impairment following acute exposure (25). A pharmacodynamic mechanism for the development of tolerance to cannabis impairment has been described. A double-blind, randomized, placebo-controlled crossover study assessed how cannabis affected the brain in occasional and chronic cannabis users following acute cannabis or placebo dosing. In occasional users, cannabis induced significant neurometabolic alterations in reward circuitry, decreased functional connectivity, and increased striatal glutamate concentrations, along with increases in subjective high and decreases in sustained attention. Similar changes were not observed in chronic users, suggesting reduced reward circuitry responsiveness to cannabis intoxication in chronic users (2, 3).

Data on frequency, dose, and duration-dependent responses are required to study the reduction in tolerance. Current data are limited and do not enable studying the partial or complete loss of cannabis effects over time (3, 25).

## Low Adherence to Chronic Cannabis Administration

Adherence to therapeutic regimens is a major challenge to maximizing the beneficial effects of chronic drugs. Almost half of the chronic medical cannabis users may stop using the drug for various reasons. Loss of effect, side effects, and lower patient engagement are three major explanations for stopping drug use.

Of the older adults receiving cannabis treatment for various symptoms such as chronic pain and sleep difficulties, only 58% continued to use it after 6 months, with one-third of them reporting adverse events (28). A retrospective, population-based cohort study using the drug administration data of 5,452 new users showed that only 18% of patients used cannabinoids at 1 year. The median use duration was 31 days. The use duration varied with the type of cannabinoid medication, age of patient, socio-economic status, and diagnosis (29). A study of patients licensed to use medical cannabis showed that 20% of them did not adhere to medical cannabis use. The variables associated with adherence were illness type, cancer vs. non-cancer, and adverse effects. Patient–physician relationship and degree of satisfaction from medical cannabis use were important parameters for adherence (30).

## Overcrowded Cannabis Products Market: Need for Market Differentiators

Opening up the cannabis market led to an increase in number of companies manufacturing and selling cannabis products. This highly competitive market requires market differentiators, but the challenges in product standardization and variability in products and user-dependent factors make the generation of differentiators difficult. Narrow revenue margin is a major risk for many of these companies.

Following the legalization of its use, the prices of cannabis fell steadily and proportionally at the processor and retailer levels. An analysis of the effects of cannabis legalization on its use and prices showed that in the United States, legalization increased the frequency of use among adults and reduced its prices (31). The lack of an effective overarching federal regulatory structure and the rapidly growing cannabis industry raised the need for ways to maximize company profits (32). The retail and wholesale prices of multiple product types could be maintained at the ratio of roughly 3:1 after some initial fluctuations (11). The Herfindahl–Hirschman index (HHI) for processors and retailers after the legalization of cannabis in Washington state showed the cannabis market to be highly competitive at the processor level and less competitive for retail markets at the county level (11).

The cannabis cost per pound is also sensitive to the average batch size and testing failure rates. The loss of cannabis when a batch that fails testing is destroyed accounts for a larger share of the total testing costs. Testing standards also affect the cost of supplying licensed cannabis under similar testing regimes (33).

An analysis of 110 million retail transactions in cannabis products showed that estimating the potency data for edibles and identifying the extract subtypes are relevant to prices. Extracts accounted for 28% of sales. Of the extracts categorized by subtype, half were identified as “dabs” and the other half were identified as “cartridges.” The price per 10 mg THC was higher for edibles, medium for cartridges, and lower for other cannabis flower and extracts. Solid concentrates offered the lowest priced THC from among all the extract products. High-CBD chemovars are becoming more common, but are rare in flower marijuana and among extract products (34).

## First-Generation AI Systems are Not Enough to Improve the Response to Chronic Drugs and Create Market Differentiators

First-generation AI systems are meant to improve health care. These systems largely focus on clinical decision making through big data analysis to generate evidence-based information. However, their real-world utilization is limited because most algorithms need not necessarily result in better patient outcomes (35–40).

Big data analysis is associated with biases impacting the overall results of these algorithms. The data used by first-generation systems sometimes lack in well-structured and stable training sets. First-generation systems also fail to explain the decision-making algorithms clearly (36). Concerns such as unacceptable results, difficulty in identifying the risk of unquantified biases, and the possibility of using inappropriate confounding variables make it extremely desirable to have a system that explains the algorithms more clearly (36). Many of the current algorithms used lack the ability to make clinically relevant associations. The improved accuracy that these systems seek does not necessarily represent better clinical efficacy (41). The lack of clear beneficial effects is a major obstacle to clinicians and patients adopting these systems (42).

Patient engagements wherein the patients take the responsibility of their health do not necessarily improve with the use of first-generation systems (43). The use of mobile phones to remind patients to take chronic drugs is not enough to improve adherence. A review of multiple studies on the use of mobile phones reminding patients to take anti-retroviral drugs showed a positive effect on adherence in only 41% of the studies, with only 12% improvement in retention (44).

## Second-Generation AI Systems-Based Cannabis Regimens Focus on Improved Patient Outcomes and Deal with the Inter- and Intra-subject Variability in Responses

Second-generation AI systems focus on improved patient clinical outcomes with single subjects (42). First-generation systems are designed to promote the 4P—predictive, preventive, personalized, and participatory—medicine model, providing patient autonomy (45). Second-generation AI systems add the “5th P,” progress, to improve clinically meaningful outcome in a subject-tailored manner (42). Personalized closed-loop second-generation systems can be used to improve patient responses to chronic therapies (42). By focusing on patients' clinical benefits, they ensure increased adherence to therapeutic regimens and sustainable response to chronic drugs, while dealing with the compensatory mechanisms associated with tolerance and disease progression (24, 42).

Second-generation systems implement closed-loop algorithms to improve responses as measured by clinical outcomes or reduced side effects, which are relevant parameters for patients and caregivers. To deal with big data biases, these systems implement an *n* = 1 concept in personalized therapeutic regimens. The focus of these systems is to improve the clinically meaningful endpoint of an individual subject (42).

A second-generation system is based on introduction of individualized variability signatures into an algorithm to improve the beneficial effects of chronic drugs (42). This approach can deal with drug tolerance and ensure the sustainable beneficial effects of chronic drug use. Regular fixed regimens for chronic drug administration are incompatible with the physiological variability in biological systems and may underlie the primary and secondary lack of responses to chronic drug administration (46–50). The introduction of variability into therapeutic regimens can improve the response to drugs (48–62). Intermittent dose escalations and reductions along with drug holidays improve the respose to chronic therapies (63–67). Real-world data support the beneficial effects of drug holidays and dose escalation/reduction. The use of second-generation systems enables quantifying the individualized variability patterns and implementing them through algorithms (42, 48, 49, 54).

The second-generation system version 1.0 determines the effect of introducing variability into the therapeutic regimens of subjects who have lost their responses to chronic medications by using pseudo-random number generators that introduce variability in administration times and dosage within an approved range. Ongoing clinical trials evaluate these regimens in patients with inflammatory bowel disease who have lost their response to anti-TNFs and in patients with drug-resistant epilepsy. Version 2.0 consists of a closed-loop algorithm receiving inputs based on clinical outcomes. Version 3.0 uses host- and disease-related variability patterns continuously quantified in a personalized manner and implemented through true-random number generators. The system ignores the genotypic and phenotypic parameters because the total sum of all the potential factor effects on selected outcomes are considered. The system adapts itself to the sum of all parameters via their effects on clinical outcomes (42).

## Digital Medical Cannabis Provides Market Differentiators: Advantages for Patients, Clinicians, Health Care Authorities, Payers, and Cannabis Companies

The highly competitive market of cannabis companies requires them to develop market differentiators. However, the lack of standardization in therapeutic regimens and high variability in products and patient responses are major challenges. Prices can be considered a differentiator, but their low margins do not allow companies to use them as differentiator.

Digital medical cannabis is a cannabis product controlled by a second-generation AI system applied through a user-friendly app downloaded to a cell phone. The system enables the subject to follow a personalized therapeutic regimen that increases adherence and improves patient responses to cannabis products easily by dealing with their tolerance. Digital medical cannabis provides several market differentiators for cannabis companies.

The second-generation app improves some clinically meaningful outcomes for users and hence can expect increased patient adoption. Improved patient and clinician experience with digital medical cannabis due to enhanced product effect will increase its sales and enable better pricing. The collection of real-world data on fixed dosing regimens vs. algorithm-controlled therapy use will further support the wide implementation of these systems in the cannabis market. This system can increase the savings of users and institutions by continuing the patients on medical cannabis and avoiding the need for more expensive drugs. This might also motivate health care institutions to support the use of digital medical cannabis.

**Figure 1:**
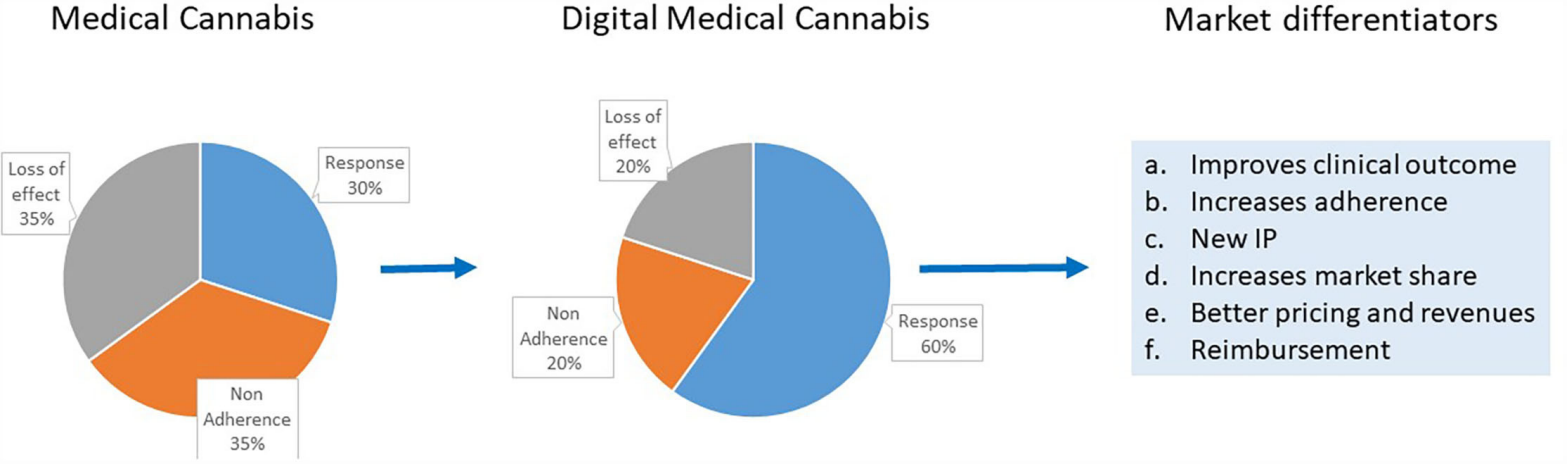
Schematic presentation of the advantages to all players in the cannabis field from digital medical cannabis use and the current medical cannabis products use. Some of the market differentiators provided by digital medical cannabis are highlighted.

Figure 1 gives a schematic presentation of the advantages of digital medical cannabis use for all players in the cannabis field.

Digital medical cannabis represents the combination of a cannabis product and a second-generation AI system to create a new intellectual property (IP). This new IP is a profound market differentiator that can help companies to increase their market share. The use of digital medical cannabis can also generate big data resources with focus on clinically meaningful endpoints and deal with the biases inherent in first-generation systems. This new big data resource type can also serve as basis for a new IP and further improvement in algorithms.

Digital medical cannabis provides advantages to all players in the health care system. While clinicians and patients can enjoy its clinical benefit, drug manufacturers can expect increased sales and the health care system can save in costs.

## Business Model for Digital Medical Cannabis: A Win-Win for Patients, Care Givers, Cannabis Companies, and the Health Care System

Digital medical cannabis maximizes the therapeutic effect of cannabis products. It provides clear benefits to end users, patients, and physicians by increasing adherence and dealing with tolerance; improves the response to a cannabis product; and reduces side effects without additional costs. Cannabis companies benefit from increased market share and revenue by having market differentiators based on improved clinical outcome. Maximizing clinical benefits enables the health care systems to save without raising the overall health care budget.

In contrast to most first-generation AI systems, second-generation systems are self-sustained without imposing additional costs. The costs associated with developing and supporting digital systems are charged not to the health care system, but to the increased revenue of drug manufacturers and savings of insurance companies.

## Regulatory Implication of Digital Cannabis Use

The digital medical cannabis version 1.0 consists of an open-loop system that does not collect or generate new data, but remains within the domains of reminders improving patient adherence. From the regulatory authorities' perspective, these systems may be exempt from all regulatory processes (68). Later versions use closed-loop regimens for personalized therapies and data collection, and need to be proved superior to the fixed dosing regimens in order to gain approval.

## Summary

Digital medical cannabis is a cannabis product with a second-generation AI system that improves patient responses to drugs by increasing adherence and dealing with the tolerance to drugs. The use of digital medical cannabis is expected to improve standardization, reduce health care costs, maximize therapeutic benefits, and increase company revenues. Ongoing trials and real-world data on the use of these systems are expected to further support the use of digital medical cannabis for improving patient health.

## Author Contributions

The author confirms being the sole contributor of this work and has approved it for publication.

## Conflict of Interest

YI is the founder of Oberon Sciences.

## Publisher's Note

All claims expressed in this article are solely those of the authors and do not necessarily represent those of their affiliated organizations, or those of the publisher, the editors and the reviewers. Any product that may be evaluated in this article, or claim that may be made by its manufacturer, is not guaranteed or endorsed by the publisher.

## Abbreviations

AI: artificial intelligence
THC: Delta9-tetrahydrocannabinol
CBD: Cannabidiol.
